# Predictors of Development and Progression of Retinopathy in Patients with Type 2 Diabetes: Importance of Blood Pressure Parameters

**DOI:** 10.1038/s41598-017-05159-6

**Published:** 2017-07-07

**Authors:** Claudia R. L. Cardoso, Nathalie C. Leite, Eduardo Dib, Gil F. Salles

**Affiliations:** 1 Department of Internal Medicine, University Hospital Clementino Fraga Filho, School of Medicine, Universidade Federal do Rio de Janeiro, Rio de Janeiro - RJ, 21941-901 Brazil; 2 Deparment of Ophthalmology, University Hospital Clementino Fraga Filho, School of Medicine, Universidade Federal do Rio de Janeiro, Rio de Janeiro - RJ, 21941-901 Brazil

## Abstract

Diabetic retinopathy (DR) is a chronic microvascular complication associated a worse prognosis. We aimed to evaluate the predictors of development/progression of DR in a cohort of 544 high-risk patients with type 2 diabetes who had annual ophthalmologic examinations over a median follow-up of 6 years. Ambulatory blood pressure (BP) monitoring and aortic stiffness by carotid-femoral pulse wave velocity were performed. Multivariate Cox survival analysis examined the independent predictors of development or progression of DR. During follow-up, 156 patients either newly-developed or worsened DR. Patients who developed/progressed DR had longer diabetes duration, higher ambulatory and clinic BP levels, higher aortic stiffness, and poorer glycemic control than patients who did not developed/progressed DR. After adjustments for baseline retinopathy prevalence, age and sex, a longer diabetes duration (p < 0.001), higher baseline ambulatory BPs (p = 0.013, for 24-hour diastolic BP), and higher mean cumulative exposure of HbA_1c_ (p < 0.001), clinic diastolic BP (p < 0.001) and LDL-cholesterol (p = 0.05) during follow-up were the independent predictors of development/progression of DR. BP parameters were only predictors of DR development. In conclusion, a longer diabetes duration, poorer glycemic and lipid control, and higher BPs were the main predictors of development/progression of DR. Mean cumulative clinic diastolic BP was the strongest BP-related predictor.

## Introduction

Diabetic retinopathy (DR) is an important cause of visual impairment and blindness among patients with diabetes^1^. Further, visual impairment as a result of diabetic retinopathy has a significant negative impact on the patient’s quality of life and their ability to successfully manage their disease^2^. The risk of DR is mainly attributable to HbA_1c_ and diabetes duration. Many studies reported that better glycemic control reduces retinopathy progression^3, 4^. However, DR can develop despite intensive glucose control, supporting that other risk factors are involved in the pathophysiology of DR. In addition, in patients with type 1 diabetes, metabolic control as measured by HbA_1c_ and disease duration account for only 11% of the risk of retinopathy, leaving 89% to other factors^5^. These data suggest that besides chronic hyperglycemia and diabetes duration, other metabolic factors, such as dyslipidemia, high blood pressure (BP), and chronic inflammation may contribute to overall risk of DR progression.

Otherwise, the influence of BP in development/progression of DR is more controversial. A prospective study reported that systolic BP (SBP) reduction may improve DR and diastolic BP (DBP) increase may worsen DR^6^. A recent meta-analysis of randomized controlled trials (RCTs), in which both type 1 and type 2 diabetic participants were included, demonstrated a beneficial effect of BP reduction in retinopathy development, but not in its progression^7^. And, up to now, the prognostic importance of ambulatory blood pressures for DR development/progression has not been investigated yet. A previous cross-sectional analysis from the Rio de Janeiro Type 2 Diabetes cohort, examining the associations between clinic and ambulatory blood pressure parameters and the presence of microvascular complications, reported that, except for DR and advanced nephropathy, ambulatory BPs are better correlates of chronic complications than clinic BPs^8^. Moreover, although ambulatory blood pressures have been demonstrated to be better predictors of total mortality and of cardiovascular morbidity and mortality than clinic BPs in type 2 diabetes and in several other clinical conditions^9–11^, no study investigated its importance as a predictor of development/progression of DR, or compared its prognostic value with that from clinic BPs.

Aortic stiffness is a proposed index of accumulated vascular risk factors burden, and it has been associated with the presence of DR^12^. We have previously demonstrated that increased aortic stiffness predicted the future development of diabetic peripheral neuropathy in type 2 diabetes^13^. Considering that microvascular diabetic complications have some common determinants, it is important to investigate whether aortic stiffness is also a prognostic marker of DR development or progression.

Therefore, we aimed to investigate the independent predictors of DR development or progression in a cohort of high-risk patients with type 2 diabetes, with special attention to the prognostic importance of ambulatory blood pressures in relation to clinic BPs, and to assess if aortic stiffness, assessed by its gold-standard method the carotid-femoral pulse wave velocity^14^, provides additional prognostic information to DR development/progression.

## Results

### Baseline characteristics and incidence of development or progression of DR during follow-up

Six hundred and forty-six patients were evaluated at baseline. Of them, 54 patients (8%) had proliferative retinopathy, 12 (1.8%) had glaucoma, and 13 patients (2%) had dense cataract and were excluded for the current analysis. During the first year of follow-up 13 patients died and 10 patients had no ophthalmologic examination, totaling 544 patients with at least two annual ophthalmologic re-evaluations (median number of ophthalmologic examinations was 5 per patient). At baseline, 144 patients (26.5%) had non-proliferative retinopathy (101 mild, 29 moderate and 14 severe). After a median follow-up of 5.8 years (range 1 to 11 years), 156 patients (28.7%) either developed or progressed DR: 77 patients (19.3% of those without DR) newly-developed DR, while 79 patients (54.9% of those with non-proliferative DR) worsened it. Table 1 outlines the characteristics of all patients and of those with and without development/progression of DR. Patients who developed/progressed DR had longer diabetes duration, used more frequently insulin, and had greater prevalences of other microvascular complications than patients who did not developed/progressed DR. Patients who developed /progressed DR had higher clinic BPs during follow-up, higher baseline ambulatory BPs, higher aortic stiffness, and poorer glycemic and lipid control than patients who did not developed/progressed DR.

**Table 1:** Characteristics of diabetic patients divided according to development or progression of retinopathy during follow-up.

Characteristics	All patients (n = 544)	Patients with development/ progression of retinopathy (n = 156)	Patients without development/progression of retinopathy (n = 388)	p-value
Age (years)	60.2 (9.5)	59.4 (9.0)	60.5 (9.6)	0.21
Male gender (%)	37.1	32.7	38.9	0.20
BMI (kg/m^2^)	29.7 (5.3)	29.6 (4.6)	29.8 (5.6)	0.80
Smoking, current/past (%)	45.2	45.5	45.1	0.99
Physical activity (%)	24.1	27.6	22.7	0.27
Diabetes duration (years)	7 (3–15)	11 (7–18)	5 (2–11)	<0.001
Chronic diabetic complications (%)				
Cerebrovascular disease	8.3	10.3	7.5	0.30
Coronary artery disease	14.7	13.5	15.2	0.69
Peripheral arterial disease	15.5	17.9	14.5	0.36
Retinopathy	26.5	50.6	16.8	<0.001
Nephropathy	28.8	43.6	22.9	<0.001
Peripheral neuropathy	27.0	39.7	21.9	<0.001
Cardiovascular autonomicneuropathy	22.2	24.4	21.2	0.45
Diabetes treatment (%)				
Metformin	88.1	87.8	88.1	0.99
Sulfonylureas	45.8	42.3	47.2	0.34
Insulin	46.1	67.3	37.6	<0.001
Dyslipidemia (%)	86.6	91.0	84.8	0.070
Statins use (%)	76.3	81.4	74.2	0.094
Arterial hypertension (%)	85.5	87.8	84.5	0.35
Number of anti-hypertensive drugs	3 (1–3)	3 (1–3)	3 (1–3)	0.87
ACE inhibitors/AR blockers (%)	92.4	92.3	92.5	0.99
Diuretics (%)	66.1	65.8	66.2	0.99
Calcium channel blockers (%)	28.4	31.6	31.0	0.92
Beta-blockers (%)	48.7	43.9	50.8	0.15
Clinic blood pressures (mmHg)				
Baseline SBP	146 (24)	147 (23)	146 (24)	0.44
Baseline DBP	84 (13)	85 (14)	84 (13)	0.45
Mean first-year SBP	140 (19)	141 (20)	139 (19)	0.18
Mean first-year DBP	79 (11)	80 (10)	78 (11)	0.12
Mean second-year SBP	140 (18)	142 (19)	139 (18)	0.033
Mean second-year DBP	78 (11)	79 (10)	77 (11)	0.025
Mean cumulative SBP	139 (16)	142 (16)	138 (16)	0.005
Mean cumulative DBP	77 (9)	79 (10)	77 (9)	0.001
Ambulatory blood pressures (mmHg)				
24-hour SBP	128 (15)	131 (16)	127 (14)	0.003
24-hour DBP	74 (10)	75 (11)	73 (9)	0.023
Daytime SBP	130 (15)	132 (16)	129 (14)	0.017
Daytime DBP	75 (10)	77 (12)	74 (9)	0.029
Nighttime SBP	120 (18)	124 (20)	118 (16)	0.001
Nighttime DBP	68 (11)	70 (11)	67 (10)	0.002
Nocturnal SBP fall (%)	9.7 (11.4)	7.9 (12.6)	10.5 (10.7)	0.029
Normal SBP dipping pattern (%)	56.1	52.3	57.7	0.29
Nocturnal DBP fall (%)	9.0 (9.6)	7.8 (9.8)	9.5 (9.5)	0.062
Normal DBP dipping pattern (%)	47.0	41.9	49.2	0.15
Laboratory variables				
Fasting glucose (mmol/L)	8.9 (3.7)	9.6 (4.5)	8.6 (3.3)	0.011
Baseline HbA_1c_ (%)	8.0 (1.9)	8.7 (2.0)	7.7 (1.7)	<0.001
Mean first-year HbA_1c_ (%)	7.6 (1.5)	8.2 (1.7)	7.4 (1.3)	<0.001
Mean second-year HbA_1c_ (%)	7.7 (1.5)	8.5 (1.7)	7.4 (1.3)	<0.001
Mean cumulative HbA_1c_ (%)	7.7 (1.3)	8.3 (1.5)	7.4 (1.2)	<0.001
Triglycerides (mmol/L)	1.6 (1.1–2.4)	1.6 (1.1–2.6)	1.6 (1.1–2.4)	0.80
HDL-cholesterol (mmol/L)	1.09 (0.30)	1.09 (0.32)	1.09 (0.29)	0.91
Baseline LDL-cholesterol (mmol/L)	3.02 (0.99)	3.14 (1.04)	2.97 (0.96)	0.063
Mean first-year LDL-cholesterol (mmol/L)	2.79 (0.85)	2.97 (0.89)	2.72 (0.82)	0.002
Mean second-year LDL-cholesterol (mmol/L)	2.66 (0.79)	2.80 (0.80)	2.60 (0.78)	0.007
Mean cumulative LDL-cholesterol (mmol/L)	2.62 (0.69)	2.77 (0.70)	2.56 (0.68)	0.001
Glomerular filtration rate (ml/min/1.73 m^2^)	90 (28)	88 (27)	92 (31)	0.16
Albuminuria (mg/24 h)	13 (7–38)	18 (8–84)	11 (6–23)	<0.001
Aortic stiffness (cf-PWV, m/s)	9.2 (2.0)	9.7 (1.8)	9.1 (2.1)	0.010
Increased aortic stiffness (cf-PWV>10 m/s, %)	21.4	27.8	18.7	0.025

Values are proportions, and means (standard deviations) or medians (interquartile range). Abbreviations: BMI, body mass index; ACE, angiotensin-converting enzyme; AR, angiotensin II receptor; SBP, systolic blood pressure; DBP, diastolic blood pressure; HbA_1c_, glycated hemoglobin; HDL, high-density lipoprotein; LDL, low-density lipoprotein; cf-PWV, carotid-femoral pulse wave velocity.

### Independent predictors of development or progression of DR

**Figure 1:**
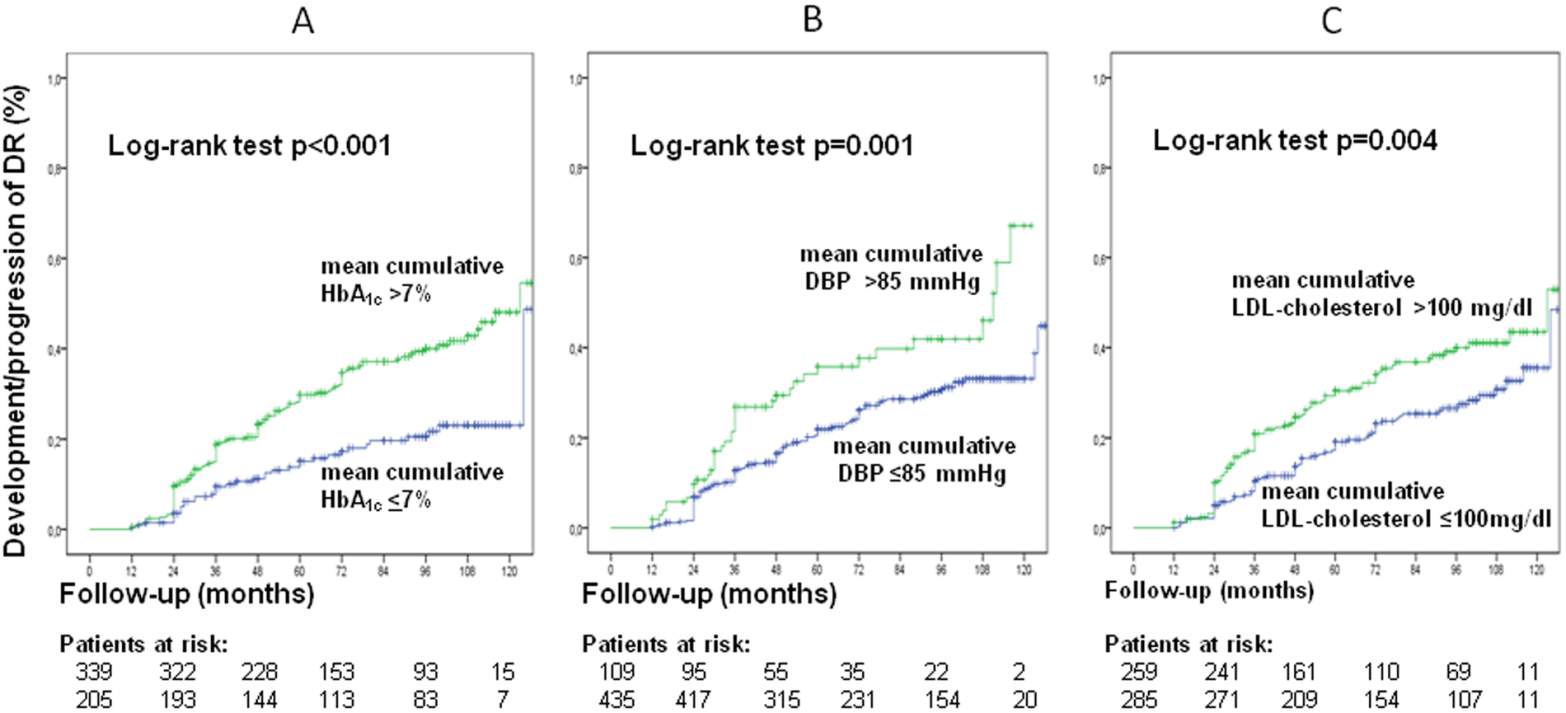
Kaplan-Meier estimation of cumulative diabetic retinopathy incidence or progression in type 2 diabetic patients grouped according to mean exposure during follow-up of HbA_1c_ (>7.0%, 53 mmol/mol, panel A), of diastolic blood pressure (>85 mmHg, panel B) and of LDL-cholesterol (>2.59 mmol/L, 100 mg/dL, panel C).

**Figure 2:**
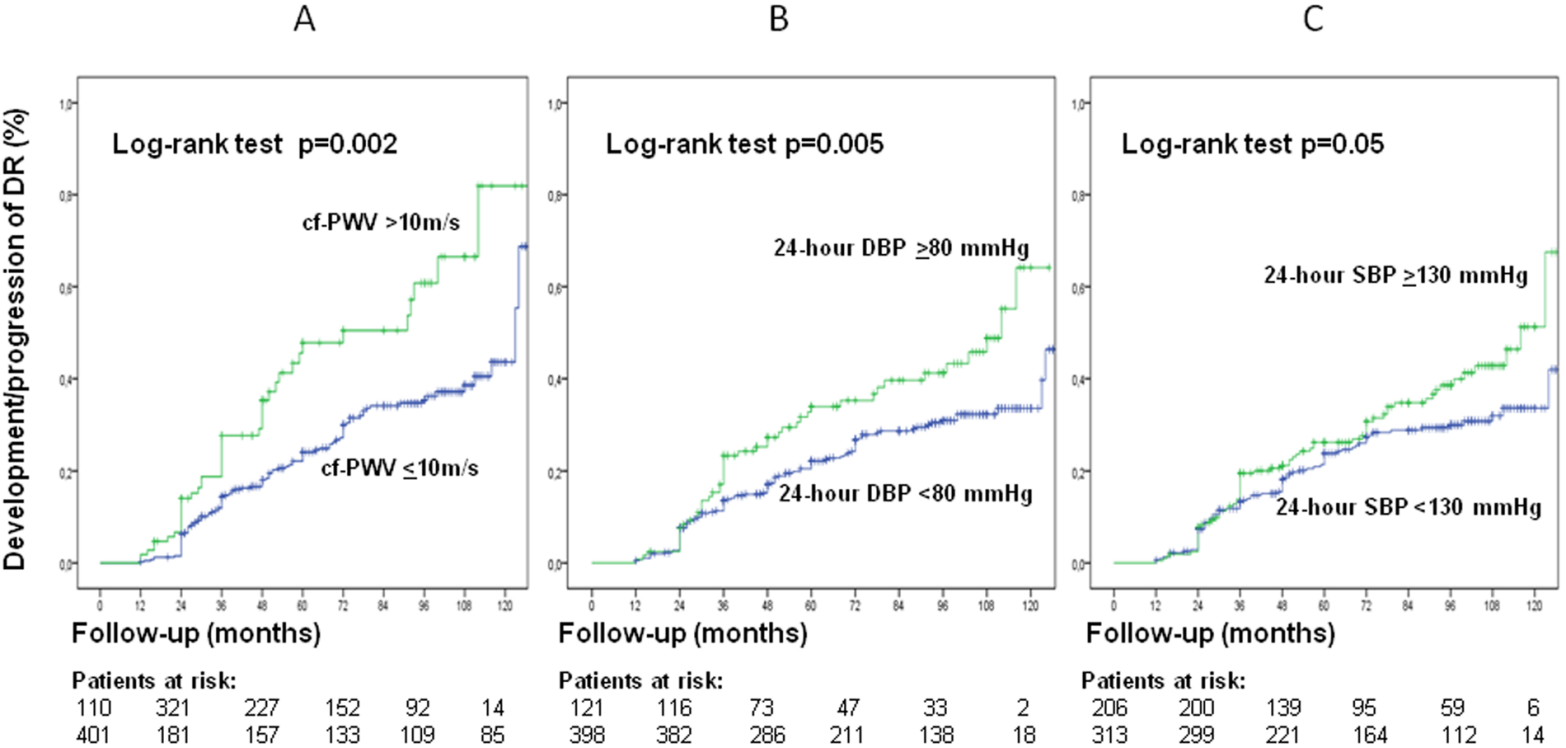
Kaplan-Meier estimation of cumulative diabetic retinopathy incidence or progression in type 2 diabetic patients grouped according to increased aortic stiffness (carotid-femoral PWV >10 m/s, panel A), ambulatory 24-hour diastolic blood pressure ≥80 mmHg (panel B), and ambulatory 24-hour systolic blood pressure ≥130 mmHg (panel C).

Table 2 presents the results of the multivariate Cox regression analysis for the independent predictors of the composite endpoint of development or progression of DR and of the separate endpoints. Longer diabetes duration and higher HbA_1c_ levels measured at any time interval during follow-up were the strongest predictors of development/progression of DR. At baseline, ambulatory BPs, but not clinic ones, were independent predictors of retinopathy development or progression. During follow-up, clinic DBPs were the main BP-related predictors of retinopathy development or progression; and in the cumulative exposure model, mean HbA_1c_ and clinic DBP were equivalent predictors of development/progression of DR. Higher LDL-cholesterol levels at baseline (with borderline significance) and in the cumulative exposure model were independent predictors of retinopathy development/progression. Regarding the separate endpoints, BP parameters were mainly predictors of new DR development, but not of DR progression (except for a borderline association with 2^nd^-year DBP), whereas HbA_1c_ levels were strong predictors of both endpoints. Increased aortic stiffness during the first-year of follow-up was a borderline significant predictor of new DR development, but not of DR progression. Kaplan-Meier analyses showed that a mean cumulative HbA_1c_ exposure >7.0%, DBP >85 mmHg and LDL-cholesterol >2.59 mmol/L (100 mg/dL) were all associated with higher risks of developing/worsening DR (Fig. 1). This was also evidenced for a baseline ambulatory 24-hour SBP ≥130 mmHg, a 24-hour DBP ≥80 mmHg and an increased aortic stiffness (cf-PWV >10 m/s) (Fig. 2).

**Table 2:** Results of multivariate Cox regression analysis for the independent predictors of the composite and separate endpoints (development and/or progression of diabetic retinopathy) measured at different time-intervals during follow-up.

Blood pressure parameters	Composite endpoint: retinopathy development/progression (n = 544 patients, 156 endpoints)			Separate endpoint: retinopathy progression (n = 144 patients, 79 endpoints)			Separate endpoint: retinopathy development (n = 400 patients, 77 endpoints)		
	HR	95% CI	p-value	HR	95% CI	p-value	HR	95% CI	p-value
**Model 1: Baseline variables**									
Diabetes duration (1 year)	1.03	1.01, 1.05	0.012	1.01	0.98, 1.04	0.59	1.04	1.01, 1.07	0.006
HbA_1c_ (1-SD)	1.33	1.13, 1.56	0.001	1.59	1.20, 2.10	0.001	1.25	1.01, 1.56	0.045
Ambulatory 24-h DBP (1-SD)	1.22	1.03, 1.46	0.024	0.98	0.74, 1.30	0.89	1.37	1.10, 1.71	0.005
LDL-cholesterol (1-SD)	1.17	1.00, 1.37	0.049	1.31	1.04, 1.67	0.025	1.11	0.90, 1.38	0.34
**Model 2: First-year variables**									
Diabetes duration (1 year)	1.03	1.01, 1.05	0.010	1.02	0.99, 1.05	0.29	1.04	1.01, 1.07	0.005
HbA_1c_ (1-SD)	1.26	1.07, 1.48	0.006	1.26	1.01, 1.60	0.048	1.25	1.01, 1.58	0.046
Clinic DBP (1-SD)	1.14	0.95, 1.37	0.15	0.96	0.70, 1.32	0.81	1.25	1.00, 1.56	0.050
Aortic stiffness (cf-PWV 1 m/s)	1.03	0.94, 1.13	0.49	0.95	0.84, 1.09	0.48	1.11	0.99, 1.24	0.073
**Model 3: Second-year variables**									
Diabetes duration (1 year)	1.03	1.01, 1.05	0.002	1.03	0.99, 1.06	0.14	1.04	1.01, 1.07	0.006
HbA_1c_ (1-SD)	1.36	1.18, 1.56	<0.001	1.30	1.05, 1.60	0.016	1.40	1.16, 1.69	0.001
Clinic DBP (1-SD)	1.27	1.06, 1.51	0.010	1.23	0.93, 1.62	0.15	1.28	1.01, 1.63	0.039
**Model 4: Cumulative variables during the whole follow-up (until censoring or endpoint occurrence)**									
Diabetes duration (1 year)	1.04	1.02, 1.06	<0.001	1.03	0.99, 1.06	0.12	1.05	1.02, 1.08	0.001
Clinic DBP (1-SD)	1.36	1.14, 1.61	0.001	1.13	0.86, 1.50	0.38	1.53	1.24, 1.90	<0.001
HbA_1c_ (1-SD)	1.30	1.10, 1.54	0.003	1.28	1.01, 1.63	0.038	1.29	1.00, 1.65	0.050
LDL-cholesterol (1-SD)	1.17	1.00, 1.39	0.057	1.29	0.98, 1.69	0.073	1.07	0.88, 1.34	0.45

Candidate variables to enter the models were the following: age, sex, BMI, smoking status, physical activity, presence of any macro- and microvascular complications, diabetes duration and classes and numbers of anti-diabetic and anti-hypertensive medications (in all models); ambulatory 24-hour systolic and diastolic blood pressures (in the baseline model); aortic stiffness (in the first-year model); and baseline, mean first-year, second-year and cumulative HbA1_c_, LDL- and HDL-cholesterol, and clinic systolic and diastolic blood pressures (in their respective models). Regardless of their significance, all models were further adjusted for age, sex and presence of diabetic retinopathy, nephropathy and peripheral neuropathy at baseline.

Abbreviations: HR, hazard ratio; CI, confidence interval; HbA_1c_, glycated hemoglobin; SD, standard deviation; DBP, diastolic blood pressure; LDL, low-density lipoprotein.

Table 3 presents the predictive strength of several BP parameters for the composite endpoint of DR development/progression and for the separate endpoints, adjusted for the other independent predictors. As a whole, clinic DBP was a stronger predictor than clinic SBP during follow-up, whereas at baseline ambulatory BPs were superior to clinic BPs. Moreover, BPs were only associated with new development of DR, but not with its progression. The strongest BP-related predictor of new DR development was mean cumulative clinic DBP during follow-up, where a 1-SD increment was associated with a 53% higher risk of DR development. On baseline ABPM, daytime and nighttime BPs were roughly equivalent in their predictive values, and the nocturnal BP fall provided no additional predictive information at all.

**Table 3:** Predictive value of several clinic and ambulatory blood pressure parameters measured at different time-intervals during follow-up for future development and/or progression of diabetic retinopathy (composite and separate endpoints).

Blood pressure parameters	Composite endpoint: retinopathy development/progression (n = 544 patients, 156 endpoints)			Separate endpoint: retinopathy progression(n = 144 patients, 79 endpoints)			Separate endpoint: retinopathy development (n = 400 patients, 77 endpoints)		
	HR	95% CI	p-value	HR	95% CI	p-value	HR	95% CI	p-value
**Systolic blood pressures (1-SD increment)**									
Baseline clinic	0.92	0.78, 1.09	0.32	0.83	0.65, 1.07	0.16	1.02	0.80, 1.28	0.90
Mean first-year clinic	0.96	0.81, 1.14	0.64	0.87	0.69, 1.14	0.35	1.01	0.79, 1.30	0.92
Mean second-year clinic	1.17	0.99, 1.38	0.070	1.13	0.87, 1.46	0.35	1.20	0.96, 1.50	0.11
Mean cumulative clinic	1.11	0.94, 1.31	0.21	1.02	0.79, 1.33	0.87	1.20	0.96, 1.48	0.10
Ambulatory 24-h	1.19	1.01, 1.40	0.037	0.98	0.73, 1.31	0.88	1.27	1.05, 1.54	0.013
Ambulatory daytime	1.16	0.99, 1.36	0.073	0.94	0.71, 1.24	0.67	1.27	1.04, 1.53	0.017
Ambulatory nighttime	1.22	1.04, 1.43	0.015	1.10	0.84, 1.45	0.48	1.28	1.05, 1.55	0.016
Nocturnal fall	0.90	0.78, 1.05	0.18	0.85	0.69, 1.06	0.15	0.94	0.76, 1.17	0.60
**Diastolic blood pressures (1-SD increment)**									
Baseline clinic	1.01	0.86, 1.18	0.94	0.90	0.70, 1.16	0.41	1.07	0.86, 1.32	0.57
Mean first-year clinic	1.14	0.95, 1.37	0.15	0.96	0.70, 1.32	0.81	1.25	1.00, 1.56	0.050
Mean second-year clinic	1.27	1.06, 1.51	0.010	1.23	0.93, 1.62	0.15	1.28	1.01, 1.63	0.039
Mean cumulative clinic	1.36	1.14, 1.61	0.001	1.13	0.86, 1.50	0.38	1.53	1.24, 1.90	<0.001
Ambulatory 24-h	1.22	1.03, 1.46	0.024	0.98	0.74, 1.30	0.89	1.37	1.10, 1.71	0.005
Ambulatory daytime	1.24	1.05, 1.48	0.012	0.97	0.74, 1.27	0.84	1.41	1.15, 1.75	0.001
Ambulatory nighttime	1.27	1.07, 1.51	0.008	1.11	0.83, 1.47	0.48	1.35	1.09, 1.69	0.006
Nocturnal fall	0.93	0.79, 1.10	0.42	0.85	0.66, 1.09	0.20	0.99	0.79, 1.26	0.96

Models were adjusted for age, sex, diabetes duration, presence of retinopathy (for analyses of the composite development/progression of retinopathy), nephropathy and peripheral neuropathy at baseline, and their respective HbA_1c_ and LDL-cholesterol levels.

Abbreviations: HR, hazard ratio; CI, confidence interval; SD, standard deviation.

## Discussion

This prospective study with a 6-year median follow-up of a relatively large sample of high cardiovascular risk patients with type 2 diabetes has three main novel findings. First, it demonstrated that at study entry ambulatory BPs were better predictors for new DR development than clinic BPs. Second, that during follow-up mean cumulative clinic DBP was the strongest BP-related predictor of DR development. Third, that aortic stiffness is not independently associated with development/progression of DR; different from what we observed for diabetic peripheral neuropathy, where an increased aortic stiffness was a predictor for its development and progression^13^. Additionally, we demonstrated the prognostic importance of higher LDL-cholesterol levels during follow-up for development/progression of DR, and confirmed the pivotal roles of poor glycemic control and diabetes duration on installation and worsening of DR. Overall, these results suggest that, in high-risk type 2 diabetic patients, DBP may have a greater influence on development of DR; and that ambulatory blood pressure monitoring should be performed routinely in the management of these patients, not only because it allows better cardiovascular risk stratification^9^, but also because it adds prognostic information regarding increased risk of DR development.

Remarkable studies have shown that more intensive glycemic and BP control reduced the onset and progression of DR in patients with type 1^15^ and type 2 diabetes^16^. However, more recent RCTs reported controversial results in type 2 diabetes, particularly regarding BP control, with some studies showing benefit to DR prevention^17^, while others showed no advantage at all^4, 18, 19^, or only small benefit for some specific ophthalmologic lesions^20^. A recent meta-analysis^7^ supported a benefit of a more intensive blood pressure control intervention with respect to 4- to 5-year incidence of diabetic retinopathy, but not to its progression. In this context, epidemiological cohort studies are still important. Some recent cohort studies reported clinic BPs as predictors of development or progression of DR^6, 21, 22^, whereas other cohorts failed in demonstrating it^23, 24^. Indeed, we did not find any predictive power of baseline clinic BPs for future development/progression of DR, but stronger predictive capacity of mean clinic BPs during on-treatment follow-up. Furthermore, we confirmed that on-treatment clinic BPs may be more important predictors to newly-development (incidence) of DR than to DR progression, as previously suggested^7^. Otherwise, as far as we know, except for a recent small study in type 1 diabetic patients^25^, this is the first study to evaluate ambulatory BPs as predictors of DR development/progression. We demonstrated that baseline ambulatory BPs are stronger predictors than clinic BPs to development/progression of DR. This finding is not unexpected, since ABPM provides several BP measurements (in general 40 to 60) during day and night, while clinic BPs were usually measured only twice during clinical attendance. Moreover, ambulatory BPs have been largely shown as superior to clinic BPs for cardiovascular risk stratification^9–11^. Overall, our findings support the increasing use of ABPM into clinical diabetes management.

Aside from well-established biochemical pathways related to chronic hyperglycemia, no other biochemical path has been conclusively demonstrated to be important for development/progression of DR^26^. In DR, there is endothelial cell injury, loss of pericytes and break-down of blood-retinal barrier, mainly due to chronic hyperglycemia. Such alterations of microvasculature lead to dysregulation of retinal perfusion, hence making eyes with DR more prone to hyperperfusion damage from hypertension^27^. Although the exact mechanism for the pathogenesis of hypertensive damage in eyes with DR is not ascertained, it has been hypothesized that an augment in BP may damage retinal capillary endothelial cells^28^. Also, studies of retinal physiology suggest a role for BP in pathological changes of DR, as well as the participation of local renin-angiotensin system^29^. BP control may avoid hyperperfusion and reduce the probability of shearing injury to the blood vessels from hypertension and, consequently, may be beneficial in preventing the development and progression of DR by reducing the damage to endothelial cells, blood vessels and surrounding tissues from hyperperfusion^7^. Otherwise, the differential influence of systolic and diastolic BP on DR development is still debatable, with some longitudinal studies showing superiority of SBP^3, 21, 22^, whereas other studies showing preponderance of DBP^6, 25, 30^. DBP reflects more peripheral vascular resistance, hence small-resistance arterial function, while SBP reflects mainly central large-artery haemodynamics. In the present study, DBPs were stronger predictors of DR development than SBPs; hence, we may speculate that, in terms of physiopathological mechanisms of DR development, small-resistance arteries alterations, with endothelial dysfunction favoring vasoconstricting over vasodilating properties, might be more important than large-artery dysfunction. This is reinforced by the small predictive importance of aortic stiffness, which also measures large-artery function.

Opposite to glycemic control, the role of lipids in the pathogenesis of DR is less clear. There is no single lipid parameter consistently found to be associated with DR incidence or progression^31^. Nonetheless, there is more evidence that links serum levels of total and LDL-cholesterol, and triglycerides to the presence of hard exudates, since retinal exudates are often due to leakage of lipids from abnormal retinal capillaries^32–36^, which is supported by our results. Most importantly, some recent RCTs suggested that treatment of dyslipidemia might prevent development of DR^4, 37, 38^. The ACCORD eye study reported a beneficial effect of fenofibrate therapy in patients who were also receiving sinvastatin^4^, whereas the Fenofibrate Intervention and Event Lowering in Diabetes (FIELD) study, a randomized study trial using fenofibrate, showed a reduction in DR progression^37^. The Steno-2 study also showed a significant reduction in the progression of DR in patients treated with statins and/or fibrates in the intensive therapy in relation to standard therapy^38^. More recently, the VADT study reported a possible interaction between intensive glycemic control and improved lipid parameters during follow-up, where a reduction in progression of DR was only observed in patients under intensive glycemic control who also improved lipid control^39^. Taking into account the above investigations, lipid-lowering medications may have an additive effect to yield better control of DR than only strict glycemic and BP control and laser treatment.

Aortic stiffness is regarded as a marker of cumulative risk factors burden on vasculature^14, 40^, however, increased aortic stiffness was not demonstrated to be an independent predictor of future development/progression of DR, different from what we had previously demonstrated for peripheral diabetic neuropathy in this same population^13^. This might suggest that, although both have common determinants, there may be differences in their physiopathological mechanisms. Although higher HbA_1c_ and blood pressure levels were common predictors of both peripheral neuropathy^13^ and DR development/progression; diabetes duration was strongly associated with DR development/progression, but not with neuropathy progression. Indeed, diabetes duration was associated with increased aortic stiffness in our cohort^12^, and it was its inclusion into the multivariable models that mostly attenuated the prognostic importance of aortic stiffness for DR development/progression. Even though, a small, marginally significant, effect of increasing aortic stiffness on DR incidence was still demonstrated here.

There are limitations of this study that warrant discussion. First, the accuracy of diabetic retinopathy grading based on clinical ophthalmologic examination without fundus photographs. In the present study, all exams were performed by the same experienced retinal specialist, and the patients with new findings on fundoscopic examination were re-evaluated in three months to confirm the findings of fundoscopy. However, it was neither compared with the accuracy of grading based on the gold standard seven-field stereo fundus photographs, nor it was possible to assess a second observer’s grading. Nonetheless, possible random missing or incorrect diagnosis of DR incidence/progression would actually underestimate the statistical significance towards the null hypothesis for the associations between risk factors and DR development/progression. Second, this study enrolled middle-aged to elderly high-risk individuals with long-standing type 2 diabetes treated on a tertiary-care center. Therefore, our findings may not be generalized to younger patients with recent-onset diabetes or treated at primary-care. Finally, it was an observational cohort study; hence, neither cause-and-effect relationships, nor physiopathological mechanisms, can be inferred, but only speculated.

In conclusion, this study demonstrated that longer diabetes duration, poorer glycemic and lipid control, and higher BPs were the main predictors of development/progression of DR. Mean cumulative clinic diastolic BP during follow-up was the strongest BP-related predictor, although at baseline ambulatory BPs were superior to clinic BPs as predictors of new DR development. These findings support the importance of performing ambulatory BP monitoring in type 2 diabetes management, not only to refine cardiovascular risk stratification, but also to predict future onset and progression of DR. Future prospective studies with intensive multifactorial management, including optimal metabolic and cardiovascular risk factors control, and with longer follow-ups may demonstrate if it is capable of preventing or delaying development/progression of DR in patients with type 2 diabetes.

## Methods

### Patients and baseline procedures

This was a prospective study, nested within the Rio de Janeiro Type 2 Diabetes Cohort Study, with 646 patients with type 2 diabetes enrolled between August 2004 and December 2008 and re-evaluated annually for DR until December 2015 in the diabetes outpatient clinic of our tertiary-care University Hospital. All participants gave written informed consent, the local Ethics Committee (School of Medicine and University Hospital Joined Committee of Research Ethics) had previously approved the study protocol (approval number: 124/04), and all the methods were performed in accordance with the Declaration of Helsinki. The characteristics of this cohort, the baseline procedures and the diagnostic definitions have been detailed elsewhere^9, 12, 13, 41^. In brief, inclusion criteria were all adult type 2 diabetic individual up to 80 years old with either any microvascular (retinopathy, nephropathy or neuropathy) or macrovascular (coronary, cerebrovascular or peripheral artery disease) complication, or with at least two other modifiable cardiovascular risk factors (hypertension, dyslipidemia or smoking). Exclusion criteria were morbid obesity (body mass index ≥40 kg/m^2^), advanced renal failure (serum creatinine >180 μmol/L or estimated glomerular filtration rate <30 ml/min/1.73 m^2^) or the presence of any serious concomitant disease limiting life expectancy. For this sub-study, those patients who at baseline had glaucoma, dense cataract, or proliferative diabetic retinopathy were also excluded, as well as those who did not have at least two annual follow-up ophthalmologic examinations. All were submitted to a standard baseline protocol that included a thorough clinical examination, a laboratory evaluation, 24-hour ambulatory BP monitoring (ABPM) and aortic stiffness assessment by carotid-femoral pulse wave velocity (cf-PWV). Diagnostic criteria for diabetic chronic complications were detailed previously^9, 12, 13, 41^. Briefly, coronary heart disease was diagnosed by clinical, electrocardiographic criteria, or by positive ischemic stress tests. Cerebrovascular disease was diagnosed by history and physical examination, and peripheral arterial disease by an ankle-brachial index <0.9. The diagnosis of nephropathy needed at least two albuminurias ≥30 mg/24 h or proteinurias ≥0.5 g/24 h or confirmed reduction of glomerular filtration rate (<60 ml/min/1.73 m^2^, or serum creatinine >130 μmol/L). Peripheral neuropathy was ascertained by clinical examination (knee and ankle reflex activities, feet sensation with the Semmes-Weinstein monofilament, vibration with a 128-Hz tuning fork, pinprick and temperature sensations and neuropathic symptoms were evaluated by a standard validated questionnaire^13^. Clinic BP was measured three times using a digital oscillometric BP monitor (HEM-907XL, Omron Healthcare, Kyoto, Japan) with a suitable sized cuff on two occasions two weeks apart at study entry. The first measure of each visit was discarded and BP considered was the mean between the last two readings of each visit. Arterial hypertension was diagnosed if mean SBP ≥140 mmHg or DBP ≥90 mmHg or if anti-hypertensive drugs had been prescribed. ABPM was recorded during 24-hour at study entry using Mobil-O-Graph, version 12 equipment (Dynamapa, Cardios LTDA., São Paulo, Brazil). A reading was taken every 15 minutes throughout the day and every 30 minutes at night. Nighttime period was ascertained for each individual patient from registered diaries. Patients were instructed to keep their routine activities during this day. Parameters evaluated were 24-hour, daytime and nighttime BPs and the nocturnal BP fall (calculated as the percentage decrease in nighttime BP in relation to daytime levels). Normal dipping pattern was defined as a nocturnal BP fall ≥10%^10^. Laboratory evaluation included fasting glycemia, glycated hemoglobin, serum creatinine and lipids. Albuminuria and proteinuria were evaluated in two non-consecutive sterile 24-hour urine collections. Aortic stiffness was evaluated by cf-PWV measurement during the first-year of follow-up, using the foot-to-foot velocity method with the Complior equipment and software (Artech-Medical, Paris, France), as previously described^12, 41^. Direct carotid-femoral distance was corrected by a 0.8 factor and a cf-PWV >10 m/s was considered as increased aortic stiffness, as recommended^40^. The patients were followed-up regularly at least 3–4 times a year and hence all had at least 2 to 4 annual HbA_1c_, serum lipids and clinic BP measurements.

### Assessment of development and progression of DR

Presence and severity of DR was determined at baseline and annually by a single retinal specialist. Following the International Clinical Diabetic Retinopathy and Diabetic Macular Edema Disease scales^42^, severity of DR was categorized into 5 stages: no retinopathy, mild non-proliferative retinopathy, moderate non-proliferative retinopathy, severe non-proliferative retinopathy and proliferative retinopathy. When there were inter-eye differences in DR severity, the eye with the severest DR was considered for DR classification. Incidence of DR was defined as having no DR signs in both eyes at baseline and having mild to severe non-proliferative DR or proliferative DR in either of the eyes at any annual examination. Progression of DR was defined as having mild non-proliferative DR at baseline and having severe non-proliferative DR, or proliferative DR or laser photocoagulation at any subsequent annual ophthalmologic examination; or as having moderate/severe non-proliferative DR at baseline and proliferative DR at any subsequent examination. All incident new DR and worsening DR cases were confirmed on a second ophthalmologic examination at least 3 months apart.

### Statistical analysis

Continuous data were described as means (SD) or as medians (interquartile range). The primary endpoint was the development or progression of DR, according to the criteria detailed before. Patients with and without DR development/progression were compared by unpaired t test (for continuous normal variables), Mann-Whitney test (for continuous asymmetric variables) and by χ^2^ test (for categorical variables). The independent predictors of the primary endpoint occurrence were examined by Kaplan-Meier estimation of its cumulative incidence during follow-up (with the predictors dichotomized at clinic meaningful cut-off values and compared by log-rank tests), and by multivariate Cox regression models. Candidate variables to enter the multivariate Cox analyses, based on biological plausibility, were the following: age, sex, body mass index (BMI), smoking status, physical activity, diabetic and anti-hypertensive treatment (number and classes of drugs), diabetes duration, macrovascular (coronary, cerebrovascular and peripheral arterial disease) and microvascular (retinopathy, nephropathy and neuropathy) diabetic complications at baseline, aortic stiffness, clinic and ambulatory BPs, HbA_1c_, HDL- and LDL-cholesterol. Four models were fitted according to the time-interval the covariates were measured: a baseline model, a first-year, a second-year and a cumulative exposure model. In the first and second-years models, BMI, clinic BPs, HbA_1c_ and lipid mean levels were updated to the values recorded during the first and second years of follow-up; and in the cumulative exposure model the mean values of these covariates were calculated from the baseline until censoring or endpoint occurrence. Ambulatory BPs were entered only into the baseline model, whereas aortic stiffness was entered into the first-year model. Regardless of their significance, age, sex and presence of DR, nephropathy and peripheral neuropathy at baseline were forced into all models. Separate analyses for new DR development and DR progression were also performed. A forward selection procedure was used to select the independent predictors with a p-value < 0.10 as the criterion to enter and to remain into the models. To assess the relative prognostic importance of each BP parameter, clinic and ambulatory BP parameters were evaluated in separate Cox models, adjusted for the same covariates of the original predictive model. Cox regression results were presented as hazard ratios (HRs) with their 95% confidence intervals (CIs); and to allow comparisons among covariates, HRs were calculated for standardized increments of 1-SD for HbA_1c_, BPs and lipids. Statistics were performed with SPSS version 19.0 (SPSS Inc, Chicago, Il., USA), and 2-tailed p-value < 0.05 was considered significant.

### Data availability statement

The Rio de Janeiro Type 2 Diabetes Cohort Study is an on-going study, and its dataset is not publicly available due to individual privacy of the participants. However, it may be available from the corresponding author on reasonable request.

## References

[1] Sivaprasad S, Gupta B, Crosby-Nwaobi R, Evans J (2012). Prevalence of diabetic retinopathy in various ethnic groups: a worldwide perspective. Surv. Ophthalmol..

[2] Sharma S, Oliver-Fernandez A, Lui W, Buchholz P, Walt J (2012). (2005) The impact of diabetic retinopathy on health-related quality of life. Curr. Opin. Ophthalmol..

[3] Stratton IM (2001). UKPDS 50: risk factors for incidence and progression of retinopathy in Type II diabetes over 6 years from diagnosis. Diabetologia.

[4] Chew EY (2014). Action to Control Cardiovascular Risk in Diabetes Eye Study Research Group. The effects of medical management on the progression of diabetic retinopathy in persons with type 2 diabetes: the Action to Control Cardiovascular Risk in Diabetes (ACCORD) Eye Study. Ophthalmology.

[5] Lachin JM, Genuth S, Nathan DM, Zinman B, Rutledge BN (2008). DCCT/EDIC Research Group. Effect of glycemic exposure on the risk of microvascular complications in the diabetes control and complications trial–revisited. Diabetes.

[6] Liu Y (2013). Glycemic exposure and blood pressure influencing progression and remission of diabetic retinopathy: a longitudinal cohort study in GoDARTS. Diabetes Care.

[7] Do DV (2015). Blood pressure control for diabetic retinopathy. Cochrane Database Syst. Rev..

[8] Cardoso CR, Leite NC, Muxfeldt ES, Salles GF (2012). Thresholds of ambulatory blood pressure associated with chronic complications in type 2 diabetes. Am. J. Hypertens..

[9] Salles GF, Leite NC, Pereira BB, Nascimento EM, Cardoso CR (2013). Prognostic impact of clinic and ambulatory blood pressure components in high-risk type 2 diabetic patients: the Rio de Janeiro Type 2 Diabetes Cohort Study. J. Hypertens..

[10] O’Brien E (2013). European Society of Hypertension Working Group on Blood Pressure Monitoring. European Society of Hypertension position paper on ambulatory blood pressure monitoring. J. Hypertens..

[11] Roush GC, ABC-H Investigators (2014). Prognostic impact from clinic, daytime, and night-time systolic blood pressure in nine cohorts of 13,844 patients with hypertension. J. Hypertens..

[12] Cardoso CR (2009). Microvascular degenerative complications are associated with increased aortic stiffness in type 2 diabetic patients. Atherosclerosis.

[13] Cardoso CR, Moran CB, Marinho FS, Ferreira MT, Salles GF (2015). Increased aortic stiffness predicts future development and progression of peripheral neuropathy in patients with type 2 diabetes: the Rio de Janeiro Type 2 Diabetes Cohort Study. Diabetologia.

[14] Laurent S (2006). European Network for Non-invasive Investigation of Large Arteries. Expert consensus document on arterial stiffness: methodological issues and clinical applications. Eur. Heart. J..

[15] The Diabetes Control and Complications Trial Research Group (1993). The effect of intensive treatment of diabetes on the development and progression of long-term complications in insulin-dependent diabetes mellitus. N. Engl. J. Med..

[16] Matthews DR, Stratton IM, Aldington SJ, Holman RR, Kohner EM, UK Prospective Diabetes Study Group (2004). Risks of progression of retinopathy and vision loss related to tight blood pressure control in type 2 diabetes mellitus: UKPDS 69. Arch. Ophthalmol..

[17] Holman RR, Paul SK, Bethel MA, Matthews DR, Neil HA (2008). 10-year follow-up of intensive glucose control in type 2 diabetes. N. Engl. J. Med..

[18] Duckworth W (2009). VADT Investigators. Glucose control and vascular complications in veterans with type 2 diabetes. N. Engl. J. Med..

[19] Sandbæk A (2014). Effect of early multifactorial therapy compared with routine care on microvascular outcomes at 5 years in people with screen-detected diabetes: a randomized controlled trial: the ADDITION-Europe Study. Diabetes Care.

[20] Beulens JW (2009). AdRem project team; ADVANCE management committee. Effects of blood pressure lowering and intensive glucose control on the incidence and progression of retinopathy in patients with type 2 diabetes mellitus: a randomised controlled trial. Diabetologia.

[21] Kawasaki R (2011). Japan Diabetes Complications Study Group. Incidence and progression of diabetic retinopathy in Japanese adults with type 2 diabetes: 8 year follow-up study of the Japan Diabetes Complications Study (JDCS). Diabetologia.

[22] Rudnisky CJ, Wong BK, Virani H, Tennant MT (2012). Risk factors for progression of diabetic retinopathy in Alberta First Nations communities. Can. J. Ophthalmol..

[23] Jin P (2015). A five-year prospective study of diabetic retinopathy progression in chinese type 2 diabetes patients with “well-controlled” blood glucose. PLoS One.

[24] Harris Nwanyanwu K (2013). Predicting development of proliferative diabetic retinopathy. Diabetes Care.

[25] Mateo-Gavira I (2016). Nocturnal blood pressure is associated with the progression of microvascular complications and hypertension in patients with type 1 diabetes mellitus. J. Diabetes Complications.

[26] Frank RN (2004). Medical progress: diabetic retinopathy. N. Engl. J. Med..

[27] Gillow JT, Gibson JM, Dodson PM (1999). Hypertension and diabetic retinopathy–what’s the story?. Brit. J. Ophthalmol..

[28] Klein R, Klein BE (2002). Blood pressure control and diabetic retinopathy. Brit. J. Ophthalmol.

[29] Sjølie AK, Dodson P, Hobbs FR (2011). Does renin-angiotensin system blockade have a role in preventing diabetic retinopathy? A clinical review. Int. J. Clin. Pract.

[30] Porta M (2001). EURODIAB Prospective Complications Study Group. Risk factors for progression to proliferative diabetic retinopathy in the EURODIAB Prospective Complications Study. Diabetologia.

[31] Chang YC, Wu WC (2013). Dyslipidemia and diabetic retinopathy. Rev. Diabet. Stud..

[32] Chew EY (1996). Association of elevated serum lipid levels with retinal hard exudate in diabetic retinopathy. Early Treatment Diabetic Retinopathy Study (ETDRS) Report 22. Arch. Ophthalmol..

[33] Klein R (2002). ARIC Group. The association of atherosclerosis, vascular risk factors, and retinopathy in adults with diabetes: the Atherosclerosis Risk in Communities study. Ophthalmology.

[34] Heine RJ, Bouter LM, Stehouwer CD, Polak BC (2002). Blood pressure, lipids, and obesity are associated with retinopathy: the Hoorn study. Diabetes Care.

[35] Ucgun NI, Yildirim Z, Kiliç N, Gürsel E (2007). The importance of serum lipids in exudative diabetic macular edema in type 2 diabetic patients. Ann. N. Y. Acad. Sci..

[36] Sasaki M (2013). Quantitative measurement of hard exudates in patients with diabetes and their associations with serum lipid levels. Invest. Ophthalmol. Vis. Sci..

[37] Keech AC (2007). FIELD study investigators. Effect of fenofibrate on the need for laser treatment for diabetic retinopathy (FIELD study): a randomised controlled trial. Lancet.

[38] Gaede P, Vedel P, Parving HH, Pedersen O (1999). Intensified multifactorial intervention in patients with type 2 diabetes mellitus and microalbuminuria: the Steno type 2 randomised study. Lancet.

[39] Azad N (2016). VADT Study Group. Association of blood glucose control and lipids with diabetic retinopathy in the Veterans Affairs Diabetes Trial (VADT). Diabetes Care.

[40] Van Bortel LM (2012). Artery Society; European Society of Hypertension Working Group on Vascular Structure and Function; European Network for Noninvasive Investigation of Large Arteries. Expert consensus document on the measurement of aortic stiffness in daily practice using carotid-femoral pulse wave velocity. J. Hypertens..

[41] Cardoso CR, Ferreira MT, Leite NC, Salles GF (2013). Prognostic impact of aortic stiffness in high-risk type 2 diabetic patients: the Rio de Janeiro Type 2 Diabetes Cohort Study. Diabetes Care.

[42] Wilkinson CP (2003). Global Diabetic Retinopathy Project Group. Proposed international clinical diabetic retinopathy and diabetic macular edema disease severity scales. Ophthalmology.

